# Multiomics analysis unveils key biomarkers during dynamic progress of IAV infection in mice

**DOI:** 10.3389/fimmu.2025.1566690

**Published:** 2025-05-22

**Authors:** Huan Lei, Yixi Xu, Hao Zhang, Bin Zhang, Wenjun Luo, Xiao Liu, Haijun Zhang, Jinming Yang, Wen Wen, Ping Wang, Shijun Xu

**Affiliations:** ^1^ State Key Laboratory of Southwestern Chinese Medicine Resources, Chengdu University of Traditional Chinese Medicine, Chengdu, China; ^2^ School of Pharmacy, Chengdu University of Traditional Chinese Medicine, Chengdu, China; ^3^ Institute of Material Medica Integration and Transformation for Brain Disorders, Chengdu University Traditional Chinese Medicine, Chengdu, China; ^4^ School of Pharmacy, Chengdu University, Chengdu, China

**Keywords:** influenza A virus, multiomics integration, disease severity prediction, L-glutamine, biomarker

## Abstract

**Introduction:**

Influenza infection is a significant threat to public health, and identifying dynamic biomarkers that influence disease progression is crucial for effective intervention.

**Methods:**

We conducted a comprehensive evaluation of physiological and pathological parameters in Balb/c mice infected with H1N1 influenza over a 14-day period. We employed the DIABLO multi-omics integration method to analyze dynamic changes in the lung transcriptome, metabolome, and serum metabolome from mild to severe stages of infection.

**Results:**

Our analysis highlighted the critical importance of intervention within the first 6 days post-infection to prevent severe disease. We identified several novel biomarkers associated with disease progression, including *Ccl8, Pdcd1, Gzmk*, kynurenine, L-glutamine, and adipoyl-carnitine. Additionally, we developed a serum-based influenza disease progression scoring system.

**Discussion:**

This study provides new insights into the molecular mechanisms underlying influenza progression and identifies potential targets for therapeutic intervention. The developed scoring system serves as a valuable tool for early diagnosis and prognosis of severe influenza.

## Background

Influenza A virus (IAV) is a negative-sense RNA virus that has emerged as a significant global public health threat, characterized by high mortality and morbidity rates (1, 2). The timely diagnosis and accurate prediction of the infection process are crucial for mitigating influenza-related mortality and severe illness. Mouse models have been instrumental in elucidating the biomarkers associated with severe IAV infections. Numerous studies have identified various disease severity biomarkers and therapeutic targets in mouse models of influenza, such as C-reactive protein, serum amyloid A, d-alanine, itaconate, spermidine, and C-X-C motif chemokine ligand 17 (3–8). However, the dynamic research on viral infection remains limited. Understanding the evolving dynamics of the host response to IAV infection provides valuable insights for developing effective interventions to improve disease outcomes and reduce mortality rates (9).

Multi-omics analysis techniques have shown significant potential in uncovering biomarkers at the transcriptional, translational, and metabolic levels during viral infections (10). These techniques have highlighted their value for clinical diagnosis and management of respiratory infections (11, 12). For instance, transcriptomics and metabolomics analyses of lung alterations on days 7 and 9 post-influenza infection in mice have identified ARG1 as a regulator of Th1 cells (13). Macro-genomic and metabolomic analyses of cecum and serum changes on days 7 and 14 post-infection have revealed indole-3-propionic acid as a protective metabolite against influenza (14). Additionally, microbiome-metabolome association analyses of oropharyngeal swab samples have demonstrated that a specific combination of three sphingolipid metabolites exhibits high diagnostic efficacy (15). Multi-omics analysis of blood has also identified serum metabolites, such as 3-ureidopropionate, polyamine, uric acid, and tryptophan, as predictors of COVID-19 severity (16, 17). However, these studies have primarily focused on fixed time points and have not yet comprehensively analyzed the dynamic changes in lung transcriptomics, metabolomics, and serum metabolomics during influenza infection.

The detection of these identified biomarkers in easily accessible and readily collectible biological samples is essential for facilitating their application in the diagnosis and management of these diseases. While numerous studies have identified influenza-associated biomarkers across a broad spectrum of sample types, including lung, bronchoalveolar lavage fluid, trachea, intestinal tissues, and oropharyngeal swabs (14, 18, 19), the clinical applicability of these biomarkers is often hindered by the invasiveness, complexity, or instability associated with their collection. In contrast, blood is the most accessible source of samples that can be used to examine the health condition of a given organism (16, 20).

To address the gap in our understanding of viral infection dynamics within murine models, our study systematically examined changes in lung transcriptomics, metabolomic characteristics, and serum metabolomics in IAV-infected mice at multiple critical time points using a multi-omics approach. We placed particular emphasis on the biological alterations occurring during the transition from mild clinical symptoms to severe infection—a pivotal phase that is crucial for predicting disease progression and regression. By employing the advanced DIABLO analytical method to integrate and correlate the aforementioned multi-omics data, we successfully identified key biomarkers associated with influenza infection, including *Ccl8*, *Pdcd1*, *Gzmk*, kynurenine, L-glutamine, and adipoyl-carnitine. Moreover, we developed an influenza progression scoring system, which enables early diagnosis of severe influenza.

## Materials and methods

### Virus

The influenza virus was generously provided by Professor Yang Falong’s team from the Southwest Minzu University. The virus was identified as the mouse lung-adapted strain A/Fort Monmouth/1/1947 (H1N1) following sequencing by Shengong Biology. The virus was propagated in 9–10 day-old SPF-grade embryonated eggs as described (21, 22). Viral potency was ascertained using a hemagglutination assay with 0.5% chicken red blood cells, and the viral titer was stored at −80°C after aliquoting at a titer of ≥2^7^.

### Lethal dose 50%

The virus solution was diluted in PBS at 10-fold increments to achieve a range of concentrations (10–^1^ to 10^-6^). Groups of mice (n = 6 per group) were intranasally inoculated with 30 μL of each viral dilution. Mice were monitored twice daily for 14 days to record survival rates (23). The LD_50_ was calculated using the Reed & Muench method (24).

### Animal experiments

Female Balb/c mice, aged 6–8 weeks and specific pathogen-free, were purchased from Beijing Huafukang Animal Co., Ltd. A total of 64 mice were randomly allocated into experimental groups (6–8 mice per group), including viral infection groups and PBS vehicle groups. Mice were housed in an environment with a 12-hour light-dark cycle, at a temperature of 24°C ± 2°C, and a relative humidity of 50% ± 5%, alongside unrestricted availability to nourishment and hydration. All animal experiments received sanction from the Animal Ethics Committee of the Industrial Antimicrobial Research Institute at Chengdu University (Approval No: SIIA20230601). All the experiments were carried out in Biosafety Level II laboratory.

After anesthesia with pentobarbital, the virus group was intranasally inoculated with a virus suspension diluted to 0.4×LD50 with PBS, with a volume of 30 μl per mouse, the vehicle group was administered an equivalent volume of PBS via the same route. Body weight was recorded daily, and clinical symptoms of the mice were monitored and scored (25). On days 4, 6, 8, 10, and 14 post-infection, blood samples were collected at sacrifice. Subsequently, the lung, thymus and spleen tissues were immediately dissected, washed in pre-cooled 4°C physiological saline, dried on filter paper, and weighed. The organ index was calculated as (wet organ weight/body weight) × 100% (25).

### Lung histopathology

After ocular bleeding and cervical dislocation, the superior lobe of the right lung was extracted, washed with PBS to eliminate blood contamination, and then immersed in 4% paraformaldehyde for fixation. After 72 hours of fixation, the tissue was dehydrated through a gradient series, was embedded in paraffin, sectioned at approximately 5 µm thickness, and stained with hematoxylin and eosin (H&E) for slide scanning and review. Pathologists conducted blind scoring based on criteria (26, 27), which included five key indicators: pulmonary edema, alveolar/interstitial hemorrhage, degree of alveolar damage or necrosis, atelectasis, and thickening of the interlobular septa, and the extent of inflammatory leukocyte permeation in the parenchyma. The scoring criteria were divided into grades 0 to 4, where 0 indicates no lesion (normal); 1 indicates mild lesions, with less than 25% of the lung involved; 2 indicates moderate lesions, with 25-50% of the lung involved; 3 indicates severe lesions, with 75% of the lung involved; and 4 indicates very severe lesions, with 75-100% of the lung involved. All histopathological results were assessed according to this standard.

### Quantitative PCR analysis

At designated times post-infection, the middle and lower lobes of the right lung lobe were harvested and preserved in liquid nitrogen for later use. Quantitative Polymerase Chain Reaction (qPCR) was employed to ascertain the comparative mRNA levels of the H1N1 virus in lung tissue of mice. Total RNA was extracted according to the instructions of the Animal Total RNA Isolation Kit (Chengdu Fuji, catalog number: R.230701), and the viral M gene was amplified using a qPCR assay kit (Saiveier, catalog number: G3337-100). Gene expression values were normalized to α-Tubulin gene expression using the 2^−ΔΔCT^ method (28). qPCR was used to detect inflammatory factors in lung tissue of mice, and the operation was the same as the determination of lung virus titer. The primer sequences are listed in **Table 1**.

**Table 1:** Sequences of the primers.

Gene name	Primer sequence
α-Tubulin-F	ATCACAGGCAAGGAGGATGC
α-Tubulin-R	GCACTGGTCAGCCAGCTT
M-F	CTTCTAACCGAGGTCGAAACGTA
M-R	GGTGACAGGATTGGTCTTGTCTTTA
IL-6-F	TAGTCCTTCCTACCCCAATTTCC
IL-6-R	TGGTCCTTAGCCACTCCTTC
TNFα-F	GTGCCTATGTCTCAGCCTCTTCTC
TNFα-R	CCGATCACCCCGAAGTTCAGTAG
IL-1β-F	CTGGTGTGTGACGTTCCCATTA
IL-1β-R	CCGACAGCACGAGGCTTT

### Enzyme-linked immunosorbent assay

Enzyme-linked immunosorbent assay (ELISA) kit was utilized to quantify the concentrations of Interleukin-6 (IL-6), Interleukin-1 beta (IL-1β) and Tumor Necrosis Factor-alpha (TNF-α) in serum of mice (Novus Biologicals (USA), article numbers: VAL604G, VAL601 and VAL609).

### Transcriptome sequencing and differential gene analysis

We collected lung tissues from vehicle and virus-infected mice at 4, 6, 8, and 10 days post-infection (n=6 per group), with the inner halves of the left lung lobes used for transcriptome analysis. Total RNA extraction and other experimental details were conducted by Novogene (Beijing, China) and RNA integrity and quantity were assessed using an Agilent 2,100 bioanalyzer. After RNA extraction, cDNA was reverse-transcribed and then refined to secure double-stranded DNA templates for the assembly of sequencing libraries. The harvested DNA was processed for end-repair, adapter attachment, and PCR amplification to produce sequencing libraries. The fabricated cDNA libraries were subjected to Illumina sequencing followed by rigorous data quality control. The size and concentration of the libraries were evaluated using Qubit 2.0 and Agilent 2,100 to ensure library quality and sequencing accuracy. Sequences were mapped to the reference genome employing the HISAT2 software, and gene expression levels were quantified with the featureCounts tool (version 1.5.0-p3).

We used the Python programming language to generate line charts depicting the expression of all genes in both the virus and vehicle groups of mice at 4, 6, 8, and 10 days post-infection. Cosine similarity, a fundamental metric for evaluating similarity between two entities based on the cosine of the angle between vectors, is widely used in fields like pattern recognition and medical diagnostics (29). Taking the stability of gene expression in the vehicle group at each time point as a reference (assuming that gene expression changes approximate a straight line) and that there is no significant difference within the group, we utilized the cosine similarity method (with a threshold of cos(θ) < 0.5) to quantify differences in gene expression trends and identify genes with significantly different expression patterns from the vehicle group, namely differentially expressed genes (DEGs). This method can objectively identify genes with significantly changed expression patterns post-infection, providing a basis for further biological analysis and interpretation.

### Functional enrichment analysis

Gene Ontology (GO) and Kyoto Encyclopedia of Genes and Genomes (KEGG) pathway assessments were executed using the Novogene online platform (https://magic.novogene.com) with R (Version 3.0.3) and the cluster Profiler package, with the threshold for statistical importance established at P < 0.05 to ensure the reliability of the identified biological processes and pathways in statistical terms.

### Lung and serum untargeted metabolomics assays

We analyzed the lung and serum metabolomes of vehicle and virus-infected mice at 4, 6, 8, and 10 days post-infection (n=6 per group). The outer halves of the left lung lobes were ground in a mortar with liquid nitrogen, then processed with precooled methanol: water solution. After incubation and centrifugation, the liquid layer was dried under vacuum, and an internal standard was added. The supernatant was used for mass spectrometry testing. Serum samples were treated similarly, with methanol added, vortexed, and centrifuged before drying under vacuum and adding the internal standard for mass spectrometry analysis.

The Q Exactive mass spectrometer and Acquity BEH C18 column (2.1 mm × 100 mm, 1.7 µm) were used for analysis. Detection was performed by scanning positive and negative ions simultaneously using an electrospray ionization (ESI) The mobile phase consisted of 0.1% aqueous formic acid (A) and acetonitrile (B), with gradient elution (0–2 min, 5-10% B; 2–5 min, 10-15% B; 5–7 min, 15-25% B; 7–22 min, 25-85% B; 22–25 min, 85% B), The flow rate was at 0.3 mL/min, column temperature of 30°C, and injection volume of 5 µL. ESI conditions were set as follows: spray voltage of ±3.5 kV, sheath gas, auxiliary gas, and sweep gas flow rates were 35, 15, and 1 arb, respectively, and the ion transfer tube temperature was 300°C. The scan mode was full scan/data-dependent secondary scan, with primary and secondary resolutions of 70,000 and 17,500, respectively, a scan range of 75-1,100 m/z, and a collision energy gradient of 20, 40, 60 eV.

The data were preprocessed using Mzmine software (version 3.4.27), including denoising, baseline correction, peak alignment, peak identification, and normalization. In addition, leveraging precise mass measurements, secondary fragments, and isotope distribution, metabolites were identified using the HMDB and ChemSpider databases, utilizing the data on exact mass, fragment spectra, and isotopic distribution.

### Differential metabolite screening

Following the DEGs screening method, cosine similarity was used to screen for differential metabolites (DMs).

### Integrated multi-Omics data analysis

The DIABLO has proven effective in integrating datasets from various biological sources and in identifying biomarkers across metabolomics, transcriptomics, and proteomics (30–33). The DIABLO R package of mixOmics (version 6.19.4) (34) was used to integrate lung transcriptome, metabolome, and serum metabolome data. Lung transcriptome data (2,203 DEGs, 47 samples) and lung metabolome data (108 DMs, 47 samples), as well as serum metabolome data (28 DMs, 47 samples) were preprocessed. Data analysis was conducted using the mixOmics R Bioconductor package (35).

### Development of influenza progression scoring system

We selected serum metabolomic features with high weight coefficients (WC) in Comp 1 and 2 to construct the formula for the influenza disease progression scoring system. The formula for the system is as follows: influenza course score = WC × metabolite A concentration + WC × metabolite B concentration. Subsequently, the serum metabolite concentration of the vehicle and virus groups at 4, 6, 8, and 10 days post-infection were substituted into the established scoring formula for fitting, to evaluate the reliability of the scoring system and quantify the relationship between metabolite concentration and disease risk.

### Statistical analysis

Data processing and analysis were meticulously conducted using GraphPad Prism 8 statistical software (version 8.0) and Origin statistical software. Results are presented as mean ± SEM. For normally distributed data, one-way ANOVA followed by Tukey’s HSD *post-hoc* test was used. For non-normally distributed data, Kruskal-Wallis H test with Dunn’s *post-hoc* test was applied. *p* < 0.05 was considered statistically significant.

## Results

### Dynamic assessment of A/FM/1/47 (H1N1) infection in Balb/c mice

**Figure 1:**
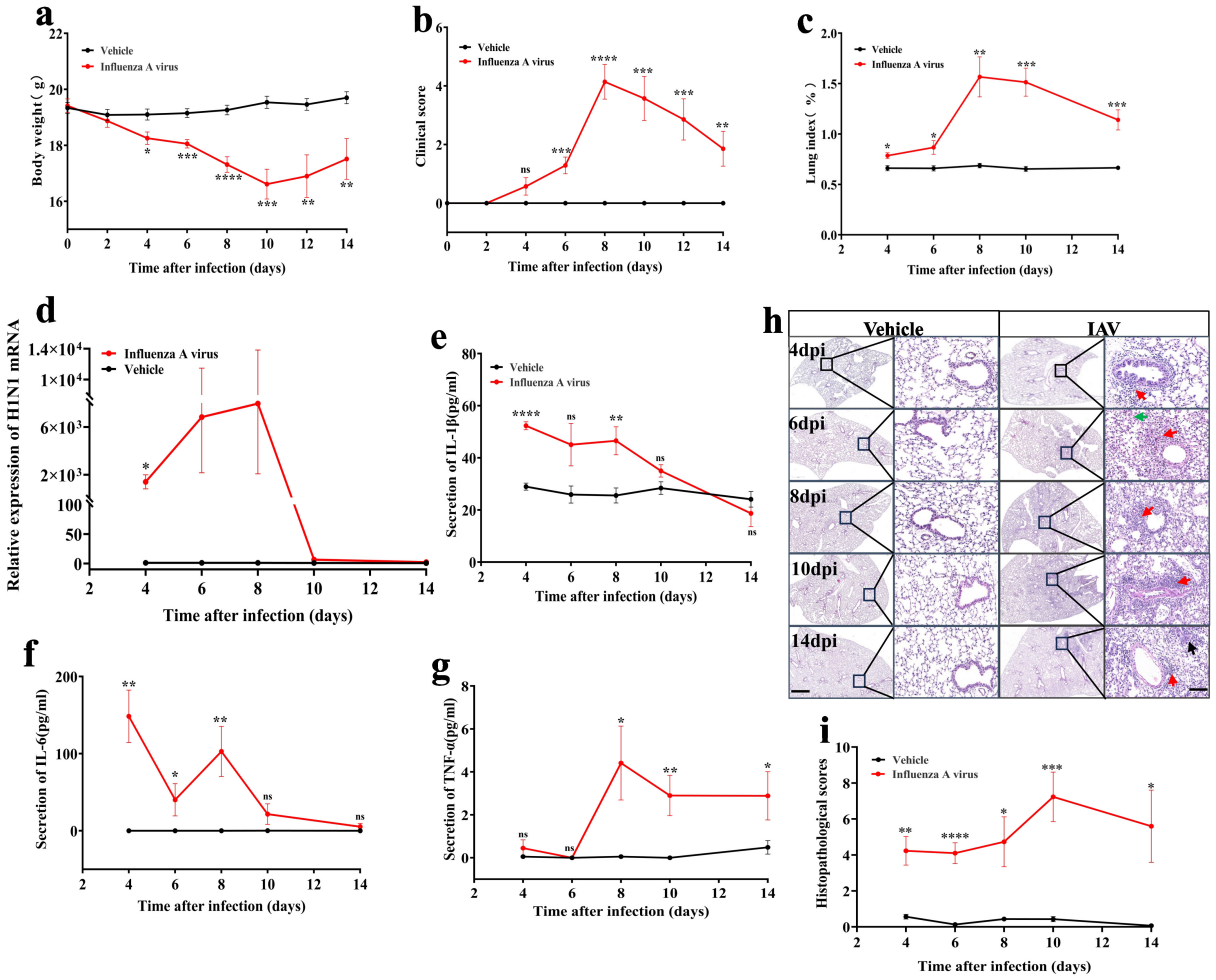
Dynamic changes in pathophysiology of mice following 14 days of IAV infection. **(a)** Changes in body weight of mice post-infection with the virus for 14 days (n = 8 for vehicle, n = 7 for IAV). **(b)** Clinical symptom scores of mice infected with influenza virus for 14 days (n = 8 for vehicle, n = 7 for IAV). **(c)** Dynamic changes in the lung index of mice (n = 6). **(d)** qPCR analysis of the viral load in lung tissue of mice over a 14-day period post-IAV infection (n = 6). **(e, f, g)** ELISA assessment of serum IL-1β, IL-6, and TNF-α levels (n=6-8). **(h)** H&E staining of lung histopathology in mice; Scale bars, 500µm and 50µm, respectively. **(i)** Statistics of histopathologic scores of mouse lungs (n = 6). Data are presented as mean ± SEM. ^*^ *P* < 0.05, ^**^ *P* < 0.01, ^***^ *P* < 0.001, ^****^ *P* < 0.0001 *vs*. the vehicle group. ns, no statistical significance.

Common indicators for assessing the pathogenicity of influenza virus in mice include weight loss, ruffled fur, shivering, hunched posture, hypothermia, and reduced activity (22), among which weight loss is the key indicator for measuring morbidity (36, 37). Initial symptoms such as piloerection and hunching were observed at 4 days post-infection (4 dpi). However, there were no notable alterations in body temperature, weight, or food intake at this stage. As the infection progressed to 6 dpi, significant physiological changes became evident. By 8 dpi to 10 dpi, infected mice exhibited approximately 15% body weight loss (**Figure 1a**), a marked increase in clinical symptom scores (**Figure 1b**), a reduction in body temperature of approximately 1.5°C (**Supplementary Figure 1a**), a substantial decrease in food consumption by around 70% (**Supplementary Figure 1b**), and the proportion of congested and edematous areas of the lungs is as high as 80% (**Supplementary Figure 1c**). In addition, compared with the vehicle group, the lung index increased by approximately 56% from 4 dpi to 8 dpi (**Figure 1c**), the thymus index decreased by about 45% at 10 dpi, and the spleen index increased by approximately 22% at 10 dpi (**Supplementary Figures 1d, e**). By 14 dpi, these indices had still not fully recovered compared with the vehicle group.

In summary, the results showed that the physiologic and pathologic indices of the mice deteriorated significantly from 6 dpi (**Figure 1**, **Supplementary Figure 1**). Therefore, it is recommended to intervene within 6 dpi to avoid progression to severe infection.

### Dynamics of viral replication and inflammatory response in mice

Viral virulence and excessive host inflammatory response are major drivers of disease progression (38, 39). Through qPCR analysis, we detected viral replication in lung tissue, revealing a substantial increase in lung viral load at 4 dpi, which escalated approximately 1000-fold compared to the vehicle group. By the 8 dpi, the viral load surged to approximately 30,000 times the baseline, followed by a gradual decline to near-normal levels between the 10 dpi and 14 dpi (**Figure 1d**).

In comparison to the vehicle group, during the early phase of infection (4 dpi), serum levels of IL-1β (*P* < 0.0001) (**Figure 1e**) and lung tissue levels of IL-1β (*P* < 0.05) (**Supplementary Figure 1f**) exhibited approximately a twofold increase, while lung tissue levels of IL-6 also doubled (*P* < 0.01) (**Supplementary Figure 1g**). Notably, serum levels of IL-6 surged by approximately 100 times (**Figure 1f**). The expression of TNF-α in serum peaked at 8 dpi, reaching about four times the level observed in the vehicle group, and remained elevated at 14 dpi (*P* < 0.05) (**Figure 1g**). Conversely, TNF-α mRNA expression levels in lung tissue increased 1.5-fold versus vehicle group at 6 dpi (*P* < 0.05), but showed significant downregulation at other timepoints (**Supplementary Figure 1h**).

The results of H&E staining of lung tissues showed significant peribronchiolar lymphocytic infiltration observed from 4 dpi (indicated by the red arrow) (**Figure 1h**). As the infection progressed to 10 dpi, there was a marked escalation in inflammatory cell presence, resulting in damage to both the alveolar and interstitial structures of the lungs, accompanied by interstitial hemorrhage (indicated by the green arrow). The pathological score reached its zenith at this stage (**Figure 1i**). Even at 14 dpi, the inflammation and damage within the lungs remained pronounced, with the emergence of fibrotic changes (indicated by the black arrow) (**Figures 1h, i)**.

### Dynamic transcriptomic changes in mouse lung tissue following IAV infection

**Figure 2:**
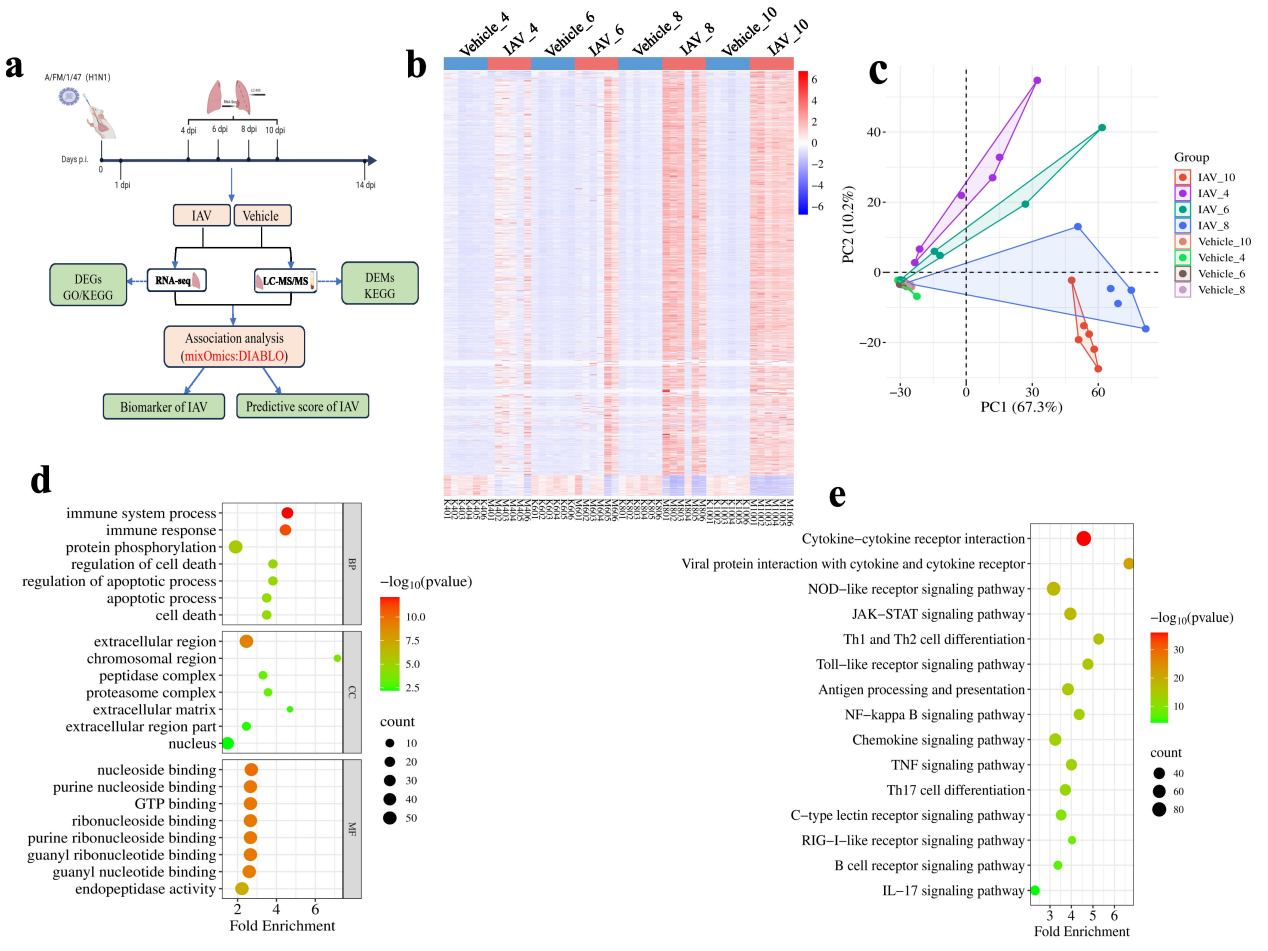
Transcriptomic changes in lung tissue of mice post-IAV infection. **(a)** Schematic of the sample collection and data analysis workflow. **(b)** Heatmap of DEGs in lung tissue at 4, 6, 8, and 10 dpi between virus-infected and vehicle groups. **(c)** PCA plot of DEGs in lung tissue at 4, 6, 8, and 10 dpi. **(d)** Bubble charts of GO functional analysis for DEGs at 4, 6, 8, and 10 dpi **(e)** Bubble charts of KEGG pathway analysis for DEGs at 4, 6, 8, and 10 dpi.

We performed transcriptional analysis of lung tissue from vehicle and viral groups of mice at 4, 6, 8,10 days post-infection and successfully extracted 56,181 clean reads after implementing strict quality control procedures (**Figure 2a**). We screened 9,158 DEGs, whose expression patterns changed significantly after viral infection, based on the FPKM values of 56,181 transcripts using cosine similarity (cos(θ) < 0.5) as a criterion. The count values of 9,158 DEGs were further screened using the criteria of no significant difference in gene expression levels in the vehicle group at different times and in samples within the group, and 2,203 DEGs were finally identified (**Figure 2b**), which showed significant expression differences at different stages of viral infection.

The outcomes of the principal component analysis (PCA) of 2,203 DEGs clearly indicated a distinct differentiation between the viral groups and the vehicle groups at various stages of infection (**Figure 2c**). Then, GO enrichment and KEGG analysis were performed on the 2203 DEGs. The biological process (BP) analysis of GO enrichment analysis indicates that DEGs are mainly enriched in areas such as “immune responses”, “protein phosphorylation” and “immune system process” (**Figure 2d**). In the cellular components (CC) analysis of GO, these DEGs are mainly located in “extracellular region”, “chromosomal region”. Regarding molecular function (MF) analysis, the results indicate that “nucleoside binding”, “purine nucleoside binding”, “GTP binding”, and “endopeptidase activity” are the most relevant items in DEGs (**Figure 2d**). KEGG pathway analysis shows that DEGs are closely related to “Cytokine-cytokine receptor interaction”, “NOD-like receptor signaling pathway”, “JAK-STAT signaling pathway” and “Thl and Th2 cell differentiation” (**Figure 2e**).

### Dynamic metabolomic changes in mouse lung tissue and serum following IAV infection

**Figure 3:**
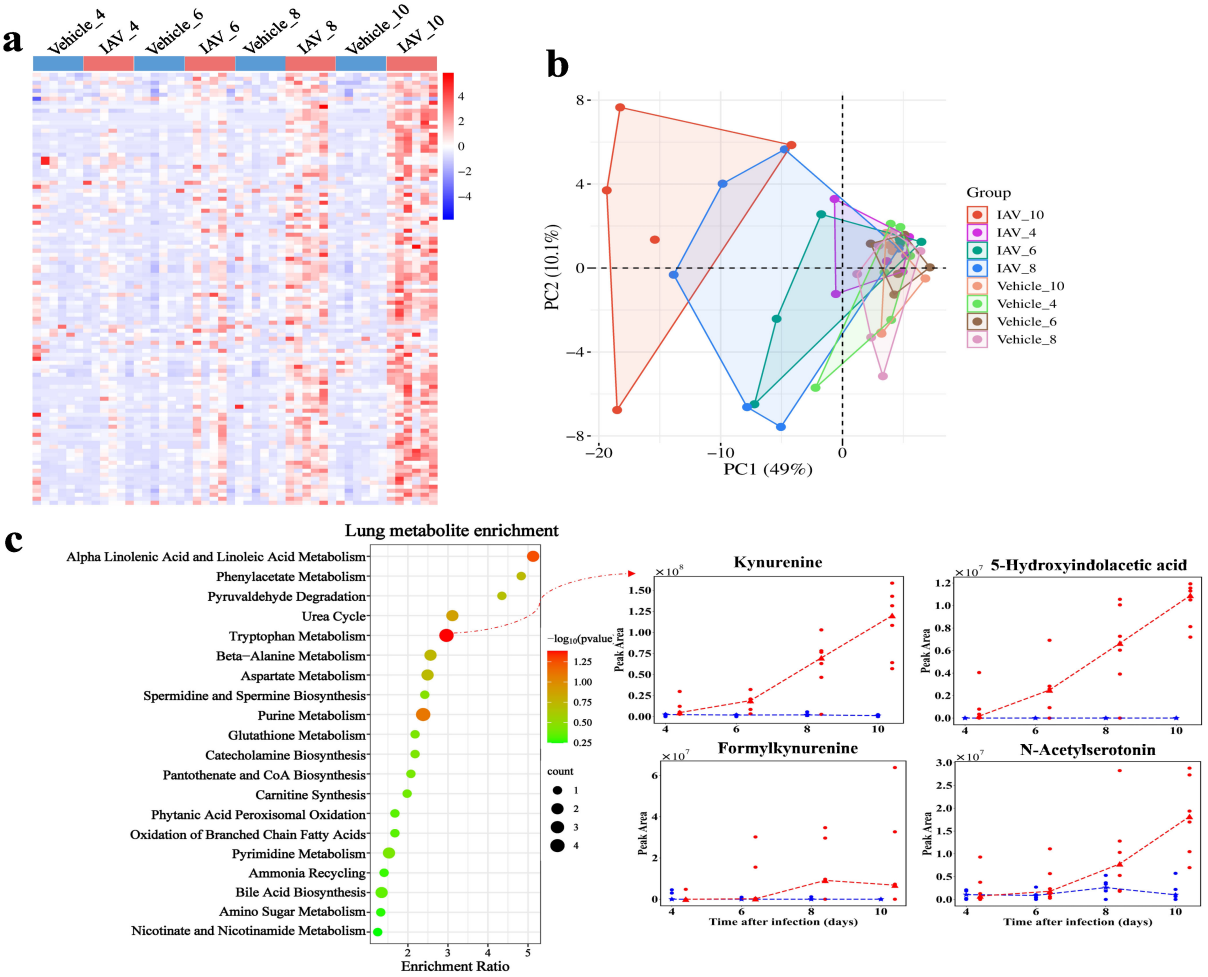
Metabolomic changes in lung of mice post-IAV infection. **(a)** Heatmap of DMs peak area content in lung tissue at 4, 6, 8, and 10 dpi between virus-infected and vehicle groups. **(b)** PCA plot of lung DMs at 4, 6, 8, and 10 dpi. **(c)** Functional enrichment bubble diagrams for lung DMs at 4, 6, 8, and 10 dpi. The line chart on the right shows the peak area concentrations of metabolites enriched in the most significant pathways.

Influenza virus infection and the associated host responses significantly alter host metabolism, which in turn affects the overall health and progression of the organism (40). We analyzed lung tissue and serum metabolism in mice at different time points after viral infection by liquid chromatography-tandem mass spectrometry (LC-MS/MS) (**Figure 2a**). This analysis resulted in the identification of 1,317 annotated metabolites in lung tissues, including 1,009 metabolites detected under positive electrospray ionization (ESI (+)) conditions and 308 under negative electrospray ionization (ESI (-)) conditions. We identified 108 DMs in lung tissue (**Figure 3a**), with PCA analysis revealing distinct separations between the virus-infected group and the vehicle group at different stages of infection (**Figure 3b**). The DMs in lung tissue were significantly enriched in the tryptophan metabolism pathway upon activation, with notable metabolites including kynurenine, 5-hydroxyindoleacetic acid, formylkynurenine, and N-acetylserotonin (*P* = 0.0414). (**Figure 3c**). Additionally, the DMs were also enriched in “alpha linolenic acid and linoleic acid metabolism”, “purine metabolism” and “beta-alanine metabolism”.

**Figure 4:**
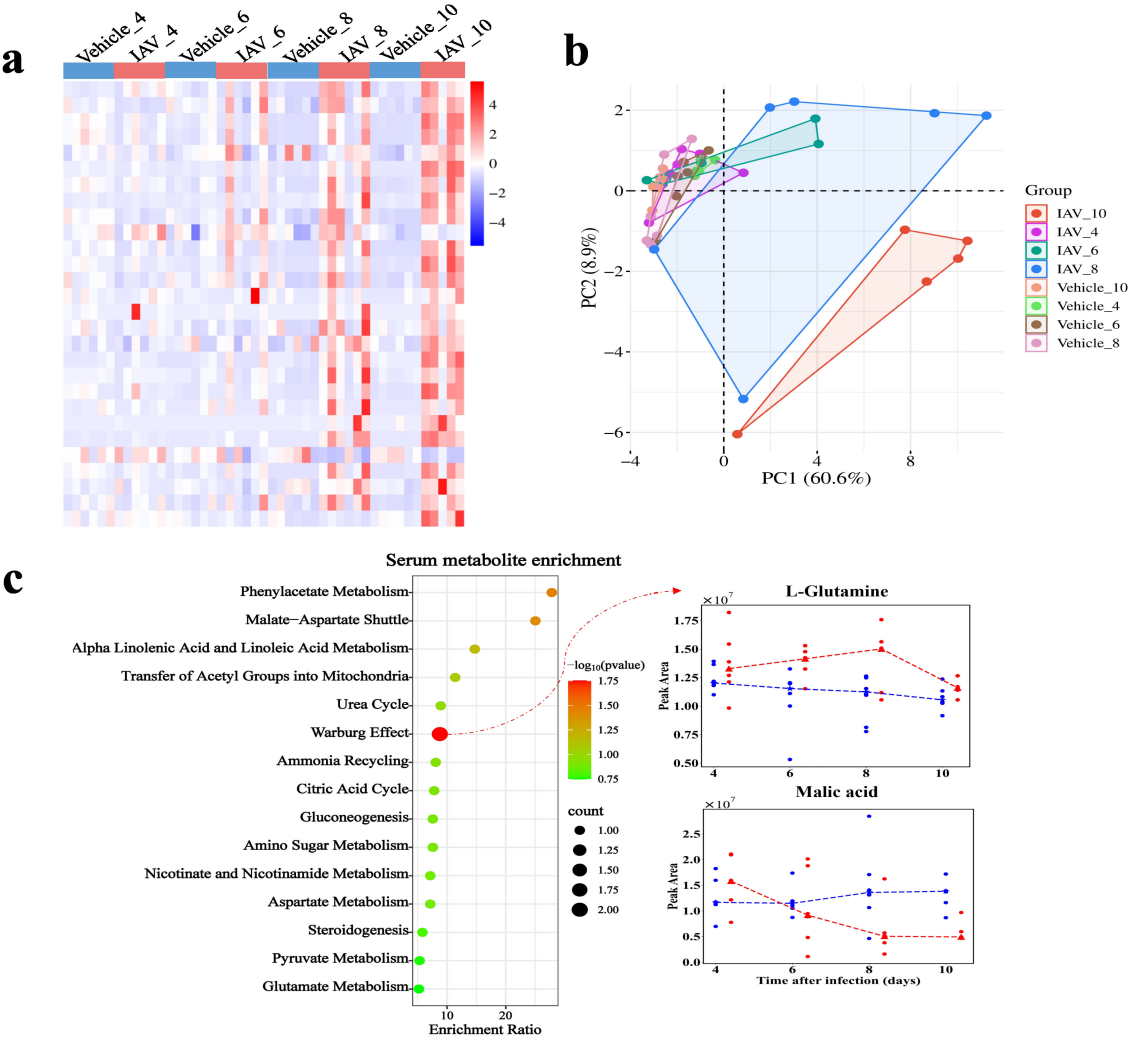
Metabolomic changes in serum of mice post-IAV infection. **(a)** Heatmap of DMs peak area content in serum at 4, 6, 8, and 10 dpi between virus-infected and vehicle groups. **(b)** PCA plot of serum DMs at 4, 6, 8, and 10 dpi between virus-infected and vehicle groups. **(c)** Functional enrichment bubble diagrams for serum DMs at 4, 6, 8, and 10 dpi. The line chart on the right shows the peak area concentrations of metabolites enriched in the most significant pathways.

In serum, we identified 1,062 metabolites, including 555 under ESI (+) and 507 under ESI (-). We identified 28 DMs in serum (**Figure 4a**). PCA demonstrated the distinction between the virus-infected groups and vehicle groups at various stages of infection in serum (**Figure 4b**). The DMs in serum were significantly enriched in pathways related to the Warburg effect, with enriched metabolites such as malic acid and glutamine (*P* = 0.0177) (**Figure 4c**). The enrichment of metabolic pathways such as “malate-aspartate shuttle”, “transfer of acetyl groups into mitochondria”, and “citric acid cycle further” indicates the host’s adaptation to increased energy demands during infection. Moreover, the enrichment of pathways like “phenylacetate metabolism”, “aspartate metabolism”, and “amino sugar metabolism” (**Figure 4c**) suggests a heightened requirement for amino acids by the host to facilitate immune responses and cellular repair processes during the infection.

### DIABLO analysis identifies dynamic biomarkers of IAV infection

**Figure 5:**
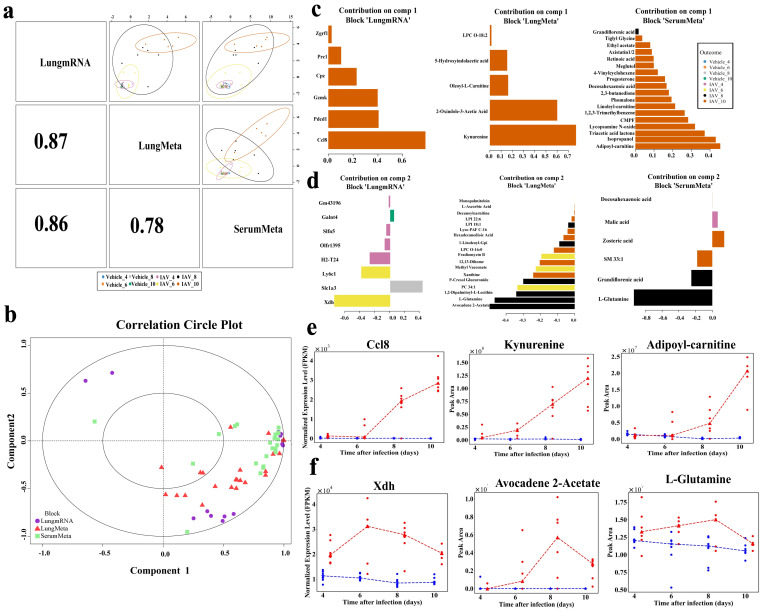
Multiomics integration and molecular characterization using DIABLO. **(a)** DIABLO scatter plot displaying the first principal components and correlations of integrated lung transcriptomic, lung metabolomic, and serum metabolomic datasets, with the upper triangle showing the first component of each omics dataset and the lower triangle showing the Pearson correlations between components. **(b)** Correlation circle plot highlighting the contribution of each selected variable in the lung transcriptomic, lung metabolomic, and serum metabolomic datasets. Important variables with greater impact on the components are positioned further from the center of the circle, while less influential variables are closer to the center. **(c)** Pyramid bar chart displaying significant features in each dataset for Component 1. **(d)** Pyramid bar chart for Component 2. **(e)** Line plots of the expression of high-weighted features in the three datasets of component 1. **(f)** Line plots of the expression of high-weighted features in the three datasets of component 2.

The sample scatterplot shows a strong correlation between lung transcriptomics and both lung and serum metabolomics, with Pearson’s correlation coefficients of 0.87 and 0.86, respectively (**Figure 5a**). Furthermore, a substantial correlation was identified between lung and serum metabolomics (Pearson’s r = 0.76), highlighting the significant interrelationships among the transcriptomic and metabolomic datasets within the samples (**Figure 5a**). Correlation circle plots further elucidated the interconnectedness of the three datasets, highlighting the contributions of key variables to each component (**Figure 5b**). Here, comp1 and comp2 reflect the key variables associated with primary and secondary biological effects, respectively. In Comp1, *Ccl8* exhibited the highest loading weight in lung transcriptomics (loading weight = 0.78), followed by *Pdcd1*, *Gzmk*, and *Cpz*. In lung metabolomics, kynurenine had the highest loading weight (loading weight = 0.77), followed by 2-oxindole-3-acetic acid, while adipoyl-carnitine was the most significant in serum metabolomics (loading weight = 0.45), followed by isopropanol and triacetic acid lactone (**Figure 5c**). In Comp2, *Xdh* exhibited the highest weight (loading weight = − 0.75), followed by Ly6c1 (**Figure 5d**). Avocadene 2-acetate (loading weight = − 0.50) and L-glutamine (loading weight = − 0.47) in lung metabolomics, L-glutamine (loading weight = − 0.93) in serum metabolomics. Notably, malic acid emerged as the sole key feature during the early stage of infection at 4 dpi. Additionally, **Figures 5e, f** illustrate the dynamic changes of the highest-weight molecular features in each dataset of comp1 and comp2, respectively.

**Figure 6:**
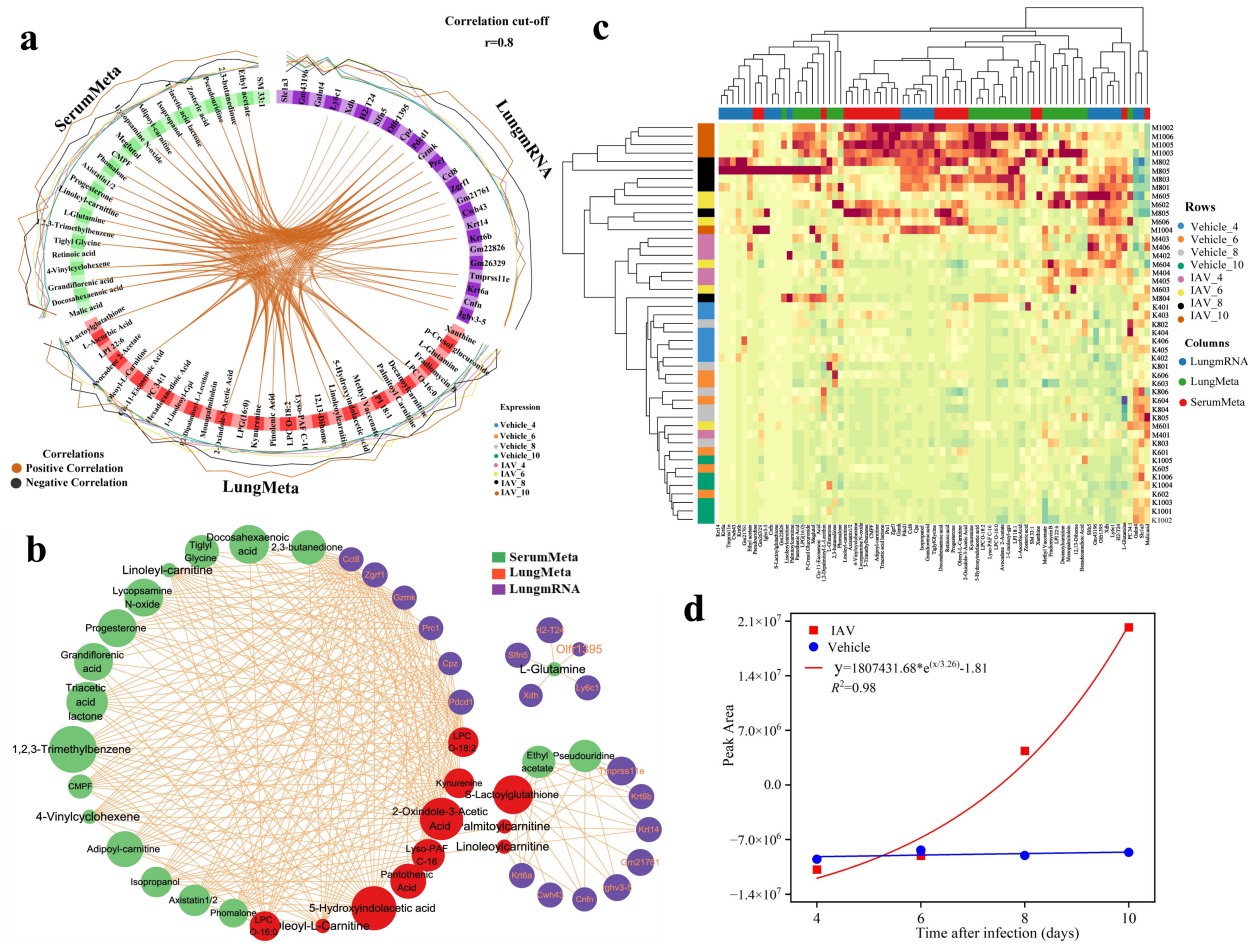
Visualization and analysis of key features from multiomics correlation. **(a)** Circos plot (cutoff: 0.8) illustrating positive (red) and negative (black) correlations among variables in the lung transcriptomic, lung metabolomic, and serum metabolomic datasets. The outer ring shows the relative abundance of each feature for each group. **(b)** Correlation network plot presenting an alternative visualization of the inter-omics variable correlations, with each color representing a type of variable. Comp1 reflects the primary source of variation in the data, Comp2 reveals secondary sources of variation. **(c)** Clustered heatmap of significant multi-omics features identified between the virus and Vehicle groups at two principal components across four infection time points, with samples represented as rows (47 samples) and each omics selected feature as columns. **(d)** Disease progression scoring curve fitting plots at different infection times.

The DIABLO analysis was ultimately depicted through circos plots (**Figure 6a**) and network interaction diagrams (**Figure 6b**). The *Ccl8* demonstrated significant positive correlations with a variety of serum and lung metabolites, including docosahexaenoic acid, tiglyl glycine, LPC O-16:0, kynurenine, and 2-oxindole-3-acetic acid (**Figure 6a**). Notably, L-glutamine exhibited considerable prominence among both serum and lung metabolites, revealing unique positive correlations with genes such as *Xdh*, *H2-T24*, *Ly6c1* and *Slfn5*, which were distinctly illustrated in the network interaction diagram (**Figure 6b**). The cluster thermogram showed the expression of 75 features identified in two principal components (**Figure 6c**).

The influenza progression scoring system formula = 0.45 × (adipoyl-carnitine concentration) + 0.43 × (isopropanol concentration) + 0.37 × (triacetic acid lactone concentration) − 0.93× (L-glutamine concentration), we incorporated the metabolite levels measured at 4, 6, 8, and 10 dpi into the curve-fitting analysis. The resulting R² value of 0.98 indicates that the scoring system accurately predicts and assesses relevant indicators, reflecting an exponential progression of influenza, suggesting that the condition had entered the severe stage when the serum score was elevated by about 2.5-fold and that the condition had entered the critical stage when the score was elevated by about 5-fold (**Figure 6d**).

## Discussion

The identification of biomarkers for IAV infection is crucial for mitigating the incidence of severe illness and mortality associated with the disease. Advances in bioinformatics and experimental methodologies have positioned comprehensive multi-omics analysis as a pivotal tool for uncovering disease markers and predicting therapeutic targets (41). DIABLO is a multi-omics integration method that maximizes the shared or relevant information among multiple omics datasets (35). As the first multivariate integrative classification method to construct predictive models, DIABLO demonstrates remarkable flexibility in handling various experimental designs (34). It excels in both classical single-time-point studies and dynamic, repeated-measures studies (42). Currently, DIABLO has been widely applied to identify key biomarkers and construct models across diverse fields (43–45). In this study, by leveraging DIABLO to integrate and analyze the dynamic changes in the lung transcriptome and metabolome as well as the serum metabolome of mice, we successfully identified specific genes and metabolites associated with influenza progression, including *Ccl8*, *Pdcd1*, *Gzmk*, kynurenine, adipoyl-carnitine, and L-glutamine.

The physiological parameters observed in mice within 14 days post-IAV infection align with findings from previous studies (46). We observed that the lung viral load and pathological damage in mice progressively increased, peaking at 8–10 dpi and then gradually declined (**Figures 1d, i**). In contrast, pro-inflammatory cytokines in the lungs and serum, including IL-6 and IL-1β, exhibited a significant initial increase at 4 dpi, followed by a sustained and gradual decrease as the infection progressed (**Figures 1e, f**), this indicates that the kinetics of inflammation and virus-induced lung damage are distinct (47). Additionally, decreased levels of TNF-α in lung tissues (**Supplementary Figure 1h**) were associated with increased tight junction permeability, which in turn exacerbated immunopathological changes and tissue remodeling in the lungs (48, 49). In the later stages of infection, elevated serum expression of TNF-α (**Figure 1g**) indicated the persistence and exacerbation of the systemic inflammatory response, suggesting that reducing serum TNF-α levels could potentially alleviate lung inflammation (50).

Our study identified a strong positive correlation between disease severity and several features in comp1, including the genes *Ccl8*, *Pdcd1*, and *Gzmk*, as well as the metabolites kynurenine and adipoyl-carnitine. *Ccl8*, a chemokine, is closely associated with the pathological degree of lung inflammation in the early stages of influenza infection (51) and serves as an indicator of lung inflammation and immune overreaction in COVID-19 (52). *Pdcd1*, involved in T cell function regulation, delays viral clearance and increases mortality in IAV-infected mice, making it a potential target for treating immune escape during IAV infection (53). *Gzmk* inhibits influenza virus replication by disrupting the importin α1/β heterodimer in the host cell’s nuclear transport complex, suggesting its potential as an antiviral therapeutic target (54). Kynurenine, a metabolite derived from host tryptophan, is positively correlated with the severity of influenza (**Figure 5e**), and a high kynurenine/tryptophan ratio is associated with adverse outcomes in influenza (55). IAV infection induces the synthesis of kynurenine, which inhibits T cell differentiation and weakens the antiviral immune response (56). Adipoyl-carnitine, an O-acylcarnitine-like metabolite of fatty acid β-oxidation (57), is a potential marker for distinguishing colorectal cancer (58), and may indicate increased energy demand in host cells during IAV infection (59).

Highly weighted features identified in Comp2 include the genes *Xdh*, *H2-T24*, and *Ly6c1*, as well as the metabolites avocadene 2-acetate and L-glutamine (**Figure 5d**). Correlation network analysis revealed a significant correlation between L-glutamine and several key genes, particularly *Xdh*, *H2-T24*, and *Ly6c1* (**Figure 6b**). *Xdh* is primarily involved in purine metabolism *in vivo*, and its upregulation during influenza virus infection leads to the accumulation of reactive oxygen species, exacerbating lung tissue damage (60). *H2-T24* is crucial for the antiviral immune response and is implicated in various immunological and inflammatory disorders (61, 62). *Ly6c1*, identified through transcriptomic analyses as a potential biomarker specific to fibroblasts, warrants further investigation into its biological functions (63). L-glutamine, an amino acid metabolite, is essential for maintaining host immune homeostasis (64, 65), and its serum levels like viral loads, peak at 8dpi and then gradually decline (**Figures 1d, f**). Viruses are known to reprogram host L-glutamine metabolism to enhance their replication and induce ferroptosis in host cells (66, 67), suggesting that targeting L-glutamine metabolism could serve as a potential antiviral strategy (68). Avocadene 2-acetate, a long-chain fatty alcohol metabolite, peaks and then declines on day 8 post-infection (**Figure 5f**), and has antiviral properties (69), indicating a possible association with viral replication.

In serum metabolite enrichment analysis, the Warburg effect (aerobic glycolysis) was significantly enriched, with related metabolites including malic acid and L-glutamine (**Figure 4f**). The Warburg effect is a common metabolic program during viral infection (70) and is a hallmark of H1N1 and COVID-19 diseases (71, 72). We found that malic acid levels gradually decreased as the infection progressed (**Figure 4f**), and high levels of malic acid have been shown to correlate with the severity of COVID-19 (73). Therefore, malic acid could serve as a marker and potential therapeutic target for monitoring the course of IAV infection. Additionally, while triacetic acid lactone and isopropanol have not received substantial attention in the context of virus infection research, their potential implications warrant further investigation. Moreover, genes such as *Cpz*, *Prc1*, and *Zgrf1*, as well as metabolites like oleoyl-L-carnitine, 5-hydroxyindoleacetic acid, and CMPF, were underweighted in the DIABLO analyses but are associated with the infection process, highlighting their potential as biomarkers.

### Limitations of the study

Despite providing novel insights into influenza progression via multi-omics integration, this study has certain limitations. Firstly, the identified biomarkers (e.g., *Ccl8*, *Pdcd1*, kynurenine, L-glutamine) and the influenza risk scoring system remain unvalidated experimentally. Future research should validate their reliability and utility using *in vitro* and *in vivo* models. Secondly, the study exclusively used female mice, neglecting potential sex-specific differences in male mice. Additionally, the study focused solely on the acute infection phase (4–10 days) without examining the recovery phase (e.g., 14 days or later), which is crucial for understanding the full infection course. Furthermore, the data presented here are from a single experiment with a limited sample size, which may affect the reproducibility and generalizability of the findings. Future work should address these limitations through comprehensive validation, extended time-point analyses, and additional experiments to ensure data reproducibility.

## Conclusions

In summary, this study reveals that the first 6 days post-infection is a critical window for intervention to prevent severe disease progression. We identified a range of potential biomarkers, including genes such as *Ccl8*, *Pdcd1*, *Gzmk*, and metabolites like kynurenine, adipoyl-carnitine, L-glutamine, which can be used for monitoring influenza risk. Additionally, the developed influenza disease progression scoring system can aid in the early diagnosis and prognosis of severe cases.

## Data Availability

The transcriptomic data presented in the study are deposited in the GEO database, accession number GSE292329. The metabolomic data for serum and lung can be found in the **Supplementary Material**.

## Supplementary material

Supplementary Figure 1 Dynamic changes in pathophysiology of mice following 14 days of IAV infection **(a)** Variations in body temperature of mice post-infection with the virus for 14 days (n = 8 for the vehicle group, n = 7 for the IAV group). **(b)** Changes in food intake of mice during the 14-day period of infection (n = 8 for vehicle, n = 7 for IAV). **(c)** Representative anatomical images of lung tissue at different stages of IAV infection in mice. **(d)** Dynamic changes in the spleen index of mice (n = 6). **(e)** Dynamic changes in the thymus index of mice (n = 6). Data are presented as mean ± SEM. ^*^ *P* < 0.05, ^**^ *P* < 0.01, ^***^ *P* < 0.001, ^****^ *P* < 0.0001 *vs*. the vehicle group. **(f, g, h)** qPCR analysis of IL-1β, IL-6, and TNF-α relative mRNA expression in lung tissue at 4, 6, 8, 10, and 14dpi (n=6-8).
