# Supplementary figures and images for: Multiomics analysis unveils key biomarkers during dynamic progress of IAV infection in mice
Source: Front Immunol. 2025 May 22;16:1566690. doi: 10.3389/fimmu.2025.1566690 (PMC12137336; doi:10.3389/fimmu.2025.1566690)

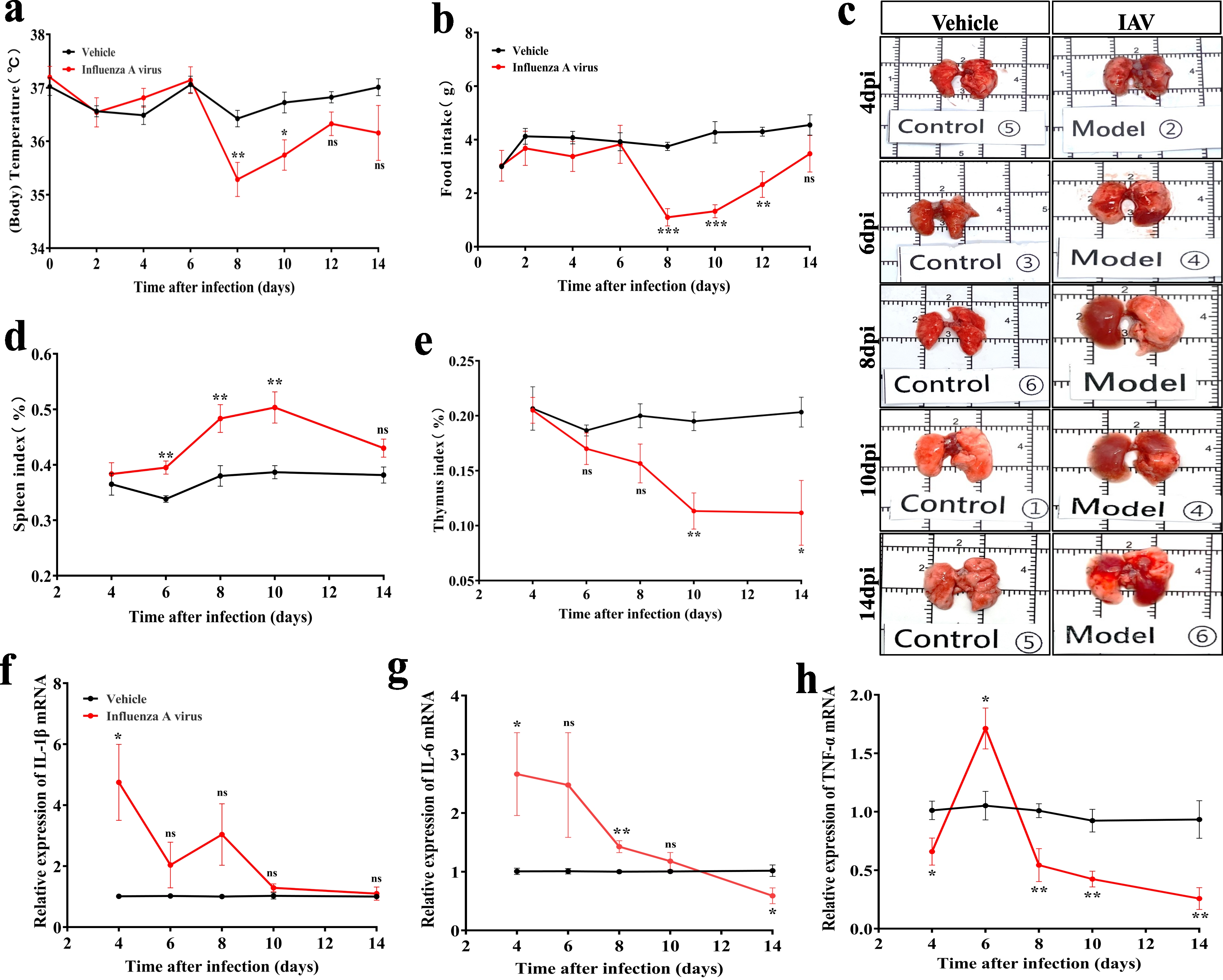

Supplement: Supplementary Figure 1 — Dynamic changes in pathophysiology of mice following 14 days of IAV infection (a) Variations in body temperature of mice post-infection with the virus for 14 days (n = 8 for the vehicle group, n = 7 for the IAV group). (b) Changes in food intake of mice during the 14-day period of infection (n = 8 for vehicle, n = 7 for IAV). (c) Representative anatomical images of lung tissue at different stages of IAV infection in mice. (d) Dynamic changes in the spleen index of mice (n = 6). (e) Dynamic changes in the thymus index of mice (n = 6). Data are presented as mean ± SEM. * P < 0.05, ** P < 0.01, *** P < 0.001, **** P < 0.0001 vs. the vehicle group. (f, g, h) qPCR analysis of IL-1β, IL-6, and TNF-α relative mRNA expression in lung tissue at 4, 6, 8, 10, and 14dpi (n=6-8). [file Image1.jpg]
